# A Cellular Basis for *Wolbachia* Recruitment to the Host Germline

**DOI:** 10.1371/journal.ppat.0030190

**Published:** 2007-12-14

**Authors:** Laura R Serbus, William Sullivan

**Affiliations:** Molecular, Cell, and Developmental Biology, University of California Santa Cruz, Santa Cruz, California, United States of America; Stanford University, United States of America

## Abstract

*Wolbachia* are among the most widespread intracellular bacteria, carried by thousands of metazoan species. The success of *Wolbachia* is due to efficient vertical transmission by the host maternal germline. Some *Wolbachia* strains concentrate at the posterior of host oocytes, which promotes *Wolbachia* incorporation into posterior germ cells during embryogenesis. The molecular basis for this localization strategy is unknown. Here we report that the *wMel Wolbachia* strain relies upon a two-step mechanism for its posterior localization in oogenesis. The microtubule motor protein kinesin-1 transports *wMel* toward the oocyte posterior, then pole plasm mediates *wMel* anchorage to the posterior cortex. Trans-infection tests demonstrate that factors intrinsic to *Wolbachia* are responsible for directing posterior *Wolbachia* localization in oogenesis. These findings indicate that *Wolbachia* can direct the cellular machintery of host oocytes to promote germline-based bacterial transmission. This study also suggests parallels between *Wolbachia* localization mechanisms and those used by other intracellular pathogens.

## Author Summary

This study focuses on *Wolbachia*, a genus of intracellular bacteria carried by insect and nematode host species. It was recently shown that *Wolbachia* carried into the human body by the host nematode Onchocerca volvulus trigger an immune response that leads to African river blindness. Findings like these raise fundamental questions of how *Wolbachia* interact with host cells to perpetuate *Wolbachia* infection. Distinct from many pathogenic bacteria, *Wolbachia* are transmitted throughout host populations primarily from females to their offspring, similar to mitochondrial inheritance. The molecular basis for this transmission strategy is unclear. Here we show that *Wolbachia* transmission is aided by a complex mechanism in egg development. Our study suggests that *Wolbachia* are transported inside the egg as cargo of molecular motors that walk along microtubule filaments. This directs *Wolbachia* to the posterior of maturing eggs, thus placing *Wolbachia* at the site where reproductive cells form during embryogenesis and ensuring *Wolbachia* integration into those cells. Furthermore, both factors intrinsic to *Wolbachia* and host molecules specifying reproductive cell fates are necessary to maximize posterior concentration of *Wolbachia* in the egg. This suggests that *Wolbachia* manipulate conserved cellular machinery in egg development to direct their transmission to the next host generation.

## Introduction

*Wolbachia* are among the most widespread intracellular bacteria, carried by an estimated 15%–76% of insect species as well as by some crustaceans, mites, and filarial nematodes [1,2]. *Wolbachia* are closely related to the *Rickettsia* family, a collection of tick-borne pathogens known for causing typhus and spotted fevers in humans. *Wolbachia* are also linked to human disease via a symbiotic relationship with pathogenic nematodes [3]. For example, the *Wolbachia*-bearing nematode Onchocerca volvulus is linked to the condition African river blindness in humans. Of the 18 million people infected by O. volvulus, nearly one million are visually impaired or already blind [4]. Recent work has implicated *Wolbachia* directly as the cause of ocular inflammation leading to river blindness [5].

The effect of *Wolbachia* infection on its host is as varied as the hosts are themselves. *Wolbachia* act as endosymbionts of some host organisms, such as the filarial nematode O. volvulus and the wasp Asobara tabida, which require *Wolbachia* in order to complete oogenesis properly [3,6]. *Wolbachia* appear to cause little phenotypic impact in certain hosts, such as in Drosophila melanogaster. In other cases, *Wolbachia* manipulate the host to their advantage. *Wolbachia* bias host reproduction to favor infected females by inducing phenotypes such as male-killing, feminization, sperm–egg cytoplasmic incompatibility, and parthenogenesis (virgin birth) [1,2]. This is thought to promote the spread of *Wolbachia* throughout host populations.

Infectious agents often spread to new hosts by becoming inhaled or ingested by that host. In the case of *Wolbachia*, however, bacterial transmission occurs within the host maternal germline [1,2]. Though *Wolbachia* are present in both male and female germlines, the bacteria are removed from sperm cysts at the end of spermatogenesis [7,8], creating a reliance upon maternal transmission. In arthropods, this maternal transmission is accomplished via incorporation of *Wolbachia* into germline precursor cells, also known as “pole cells” [9–11]. This ensures that infected females resulting from those embryos will carry bacteria in their germlines as well, thus perpetuating the *Wolbachia* transmission cycle. *Wolbachia* transmission rates have been reported at over 97% for wild-caught D. melanogaster flies, and at 100% for laboratory-reared D. melanogaster and D. simulans flies [12,13], suggesting that the pole cell–based transmission strategy is highly efficient.

How might *Wolbachia* ensure their incorporation into host pole cells? Many *Wolbachia* strains have been reported to concentrate at the posterior of mature oocytes [1,9–11,14–17]. Interestingly, the oocyte posterior pole corresponds to the location where pole cell formation takes place later in embryogenesis. For this reason, the posterior concentration of *Wolbachia* during oogenesis is thought to promote *Wolbachia* incorporation into the embryonic germline [9–11]. The cellular and molecular basis underlying this posterior *Wolbachia* localization in oogenesis is unknown to date, however.

A recent study indicated that *Wolbachia* can associate with host cell microtubules in D. melanogaster oocytes [18]. These oocytes contain an extensive network of microtubules that serves as a scaffold for cargo transport by motor proteins [19]. Up to stage 6 of oogenesis, microtubule minus ends are generally concentrated at the oocyte posterior with plus ends toward the anterior [20–22]. At stage 7, microtubules reorient such that minus ends are concentrated at the antero-lateral cortex of the oocyte, and plus ends are biased toward the posterior [23–27]. Work from D. melanogaster demonstrated that the *wMel Wolbachia* strain exhibits a microtubule-dependent concentration at the oocyte anterior from oogenesis stages 3 to 6 [18]. This anterior *wMel* localization requires the minus end–directed motor cytoplasmic dynein and the associated motor regulatory complex dynactin. However, the plus end–directed motor kinesin-1 is not required for anterior *wMel* localization [18]. These results suggest that interactions between *Wolbachia* and specific microtubule motors can direct the subcellular distribution of *Wolbachia* in oogenesis. This raises the possibility that posterior *Wolbachia* localization in late-stage oocytes may also rely upon interactions between bacteria, microtubules, and microtubule motor proteins. This also highlights *Wolbachia* as a means of understanding bacterial manipulation of host microtubules, an interaction that is considerably less well-studied than bacterial exploitation of host actin, such as in engulfment of *Salmonella* or intracellular propulsion of *Rickettsia*, *Listeria*, and *Shigella* [28,29].

How else might *Wolbachia* take advantage of the host cell to promote their posterior localization? It is possible that *Wolbachia* manipulate oocyte patterning events to their advantage. In *Drosophila*, the body axes are established via asymmetrical localization of determinant mRNAs in the oocyte [30,31]. For example, the posterior/germline determinant *oskar* (*osk*) mRNA concentrates at the oocyte posterior pole. The current model is that from stages 8 to 10A of oogenesis, kinesin-1 transports *osk* mRNA and associated Staufen (Stau) protein along microtubules toward the posterior cortex, where *osk* is translated [23–27]. Osk then initates recruitment of numerous mRNAs, proteins, mitochondria, and ribosomes to the oocyte posterior [32]. This multicomponent posterior assembly is referred to as “pole plasm”, and it functions in embryogenesis to specify posterior pole cell fates. Pole plasm is needed for posterior *wMel* localization in embryos [9]. Perhaps *Wolbachia* require posteriorly enriched substrates such as *osk*-induced pole plasm to establish their posterior localization in oogenesis as well.

This study addresses how *Wolbachia* posterior localization is achieved by examining the roles of microtubules, motor proteins, pole plasm assembly, and *Wolbachia*. Our findings indicate that during mid- to late oogenesis, kinesin-1 transports *wMel Wolbachia* toward the posterior cortex where pole plasm components mediate posterior *wMel* anchorage. The functions of kinesin-1 and pole plasm contribute independently to posterior *Wolbachia* localization. Furthermore, *wMel* can direct its localization to the oocyte posterior pole, unlike the homogeneously distributed *wRi Wolbachia* strain carried by D. simulans. This distinction between posteriorly concentrating and evenly dispersed *Wolbachia* strains may be due to different abilities of those strains to interact with posterior pole plasm.

## Results

### *Wolbachia* Concentrate at the Oocyte Posterior Pole in Mid- to Late Oogenesis

**Figure 1:**
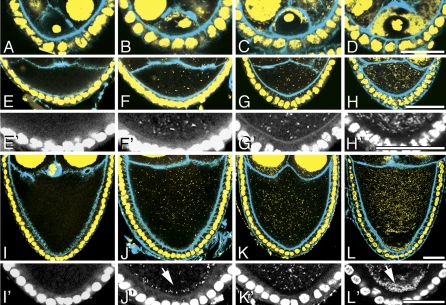
Localization of *Wolbachia* in *Drosophila* Oocytes (A–L) Oocytes from D. melanogaster and D. simulans are shown, posterior end down. Phalloidin (cyan) indicates actin, while propidium iodide (yellow) labels *Drosophila* and *Wolbachia* DNA. (E'–L') Expanded views of the oocyte posterior show propidium iodide only. Arrows indicate posterior concentrations of *wMel* puncta. Panel rows, top to bottom: (A–D) stage 5, (E–H) stage 8, (E'–H') stage 8 posterior, (I–L) stage 10A, (I'–L') stage 10A posterior. Panel columns, left to right: (A, E, E', I, I') uninfected D. melanogaster, (B, F, F', J, J') *wMel* in D. melanogaster, (C, G, G', K, K') *wRi* in D. simulans, (D, H, H', L, L') *wMel* in D. simulans. (D) bar = 12.5 μm. (H, H', L, L') bars = 25 μm.

To understand the basis for *wMel* incorporation into embryonic pole cells, ovaries were stained with propidium iodide. This showed *wMel* to be anteriorly concentrated in stage 3–6 oocytes (Figure 1A and 1B) and homogeneously distributed in stage 7–9 oocytes (Figure 1E, 1E', 1F, and 1F') [18]. From late stage 9 to stage 12, a subset of *wMel* bacteria concentrated at the oocyte posterior cortex (Figure 1I, 1I', 1J, and 1J'; Table 1) [10]. *wMel* posterior localization persisted through early embryogenesis, facilitating *wMel* incorporation into the pole cells (Figure S1) [9–11]. Thus, concentration of *wMel* at the posterior of late stage oocytes promotes germline-based transmission of *wMel*.

**Table 1:**
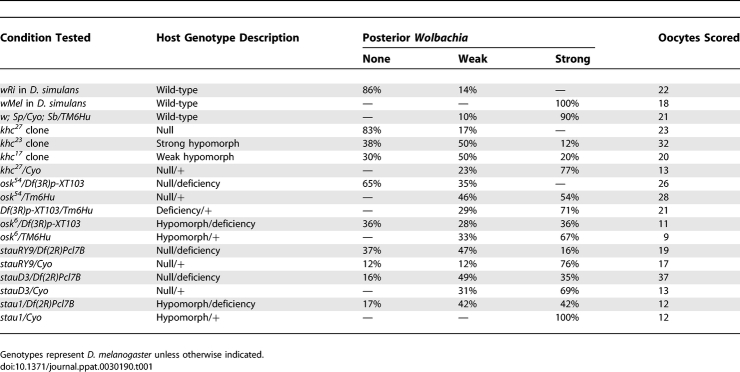
*Wolbachia* Localization in Late Stage 9 and Stage 10A Oocytes

### Directed Transport by Kinesin-1 Is Important for Posterior *Wolbachia* Localization

**Figure 2:**
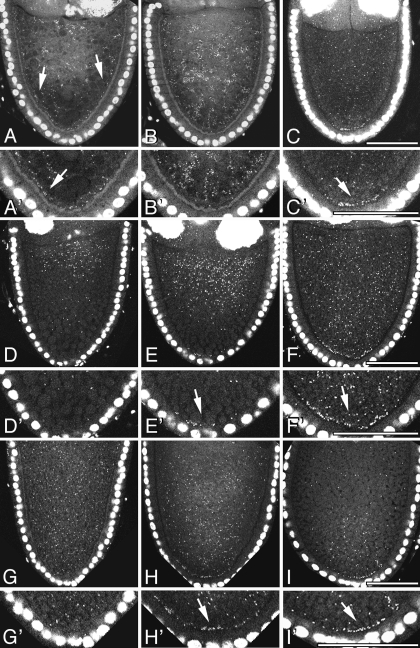
Effect of Microtubule, Kinesin-1, and *osk* Disruptions on *wMel* Posterior Localization Stage 10A *wMel*-infected oocytes are shown with propidium iodide labeling. (A–I) Full-size images are accompanied by (A'–I') corresponding expanded views of the oocyte posterior pole. Conditions shown: (A, B) colchicine-DMSO-treated, (C) DMSO-treated, (D) *Khc^27^*, (E) *Khc^23^*, (F) *Khc^27^/+*, (G) *osk^54^/oskDf^(3R)p-XT103^*, (H) *osk^54^/+*, (I) *oskDf^(3R)p-XT103^/+*. Arrows indicate enrichment of *wMel* at the (A) lateral and (A', C', E′, F', H', I') posterior cortex of the oocyte. Scale bars = 25 μm.

The redistribution of *wMel* from the oocyte anterior to posterior suggests that an active localization mechanism is involved. To test a role for microtubule-based transport in posterior *wMel* localization, oocytes were treated with colcemid and colchicine. Some colcemid-treated oocytes exhibited *wMel* at both the lateral and posterior cortex (*n* = 7 of 15 cases; Figure 2A and 2A'), while others displayed a non-cortical, homogeneous distribution of *wMel* throughout the cytoplasm (*n* = 8 of 15 cases; Figure 2B and 2B'). Colchicine-treated oocytes displayed similar broad cortical or homogeneous *wMel* localization (*n* = 13 of 20 and *n* = 5 of 20 cases, respectively). This differed from control oocytes that mainly exhibited posterior *wMel* localization (19 of 22 cases; Figure 2C and 2C'). These data indicate that microtubules are required for focused posterior localization of *wMel*.

A role for microtubules in *wMel* localization implies that a posteriorly directed microtubule motor such as kinesin-1 is involved. To determine if kinesin-1 participates in *wMel* posterior localization, we created germlines mutant for the *Kinesin heavy chain* (*Khc*) gene [23,27,33,34]. *Khc^27^* oocytes, null for kinesin function, showed normal anterior *wMel* localization during early stages (Figure S2). However, stage 10A *Khc^27^* oocytes exhibited abnormal *wMel* distribution, with *wMel* absent from the posterior cortex in 83% of oocytes (Figure 2D, 2D', 2F, and 2F'; Table 1). *wMel* was also strikingly depleted from the posterior half of *Khc^27^* oocytes (Figure 2D and 2F). Thus, kinesin-1 is important to both localize *wMel* to the posterior cortex and redistribute *wMel* into the posterior region.

The role for kinesin-1 in *wMel* posterior localization may reflect a direct or indirect *Wolbachia* localization mechanism. One possibility is that kinesin-1 transports *wMel* to the posterior as a cargo. However, kinesin-1 also drives bulk cytoplasmic streaming during mid- to late oogenesis [27,35,36]. Perhaps streaming currents sweep *wMel* passively toward the posterior cortex. To test a requirement for streaming in *wMel* localization, we examined oocytes carrying the hypomorphic mutations *Khc^17^* and *Khc^23^*. These alleles give rise to streaming-capable and streaming-deficient oocytes, respectively [27]. Posterior *Wolbachia* were exhibited by 70% of *Khc^17^* mutant oocytes and 62% of *Khc^23^* mutant oocytes (Figure 2E; Table 1). The similarity of posterior *wMel* localization in these *Khc* mutants suggests streaming is not needed for posterior *Wolbachia* localization. Rather, as both *Khc^17^* and *Khc^23^* oocytes retain some kinesin-1 function [27,37], these results indicate that *wMel* is transported toward the posterior as a cargo of kinesin-1.

### Pole Plasm Mediates Posterior Concentration of *Wolbachia*

A dependency of *wMel* on kinesin-1 for its posterior localization in oogenesis suggests *wMel* may rely on the kinesin-1 cargoes *osk* mRNA and Stau as well. Perhaps *wMel* hitchhikes to the oocyte posterior as a passenger on *osk*/Stau messenger ribonucleoprotein particles (mRNPs). Alternatively, *wMel* may require *osk*-induced pole plasm for efficient anchorage to the oocyte posterior cortex. To test these possibilities, *osk* and *stau* were disrupted with maternal-effect mutations. The majority of these mutant oocytes exhibited depletion or absence of *wMel* from the posterior cortex compared to wild-type (Figure 2G–2I, 2G'–2I'; Table 1), indicating that *osk* and *stau* gene products are important for efficient posterior *wMel* localization. Furthermore, *osk* and *stau* mutant oocytes lacking posteriorly concentrated *wMel* still exhibited a homogeneous bacterial distribution throughout the cytoplasm, differing sharply from the anterior *wMel* concentrations seen in *Khc^27^* oocytes (compare Figure 2D to 2G). This suggests that kinesin-1 can transport *wMel* into the posterior half of the oocyte independently of *osk*/Stau mRNPs. However, kinesin-1 is insufficient to drive robust *wMel* concentration at the posterior cortex in oocytes with disrupted pole plasm (Figure 2G and 2G'; Table 1). This suggests that pole plasm is important for posterior *wMel* anchorage.

### Kinesin-1 and Pole Plasm Contribute Independently to Posterior *Wolbachia* Enrichment

**Figure 3:**
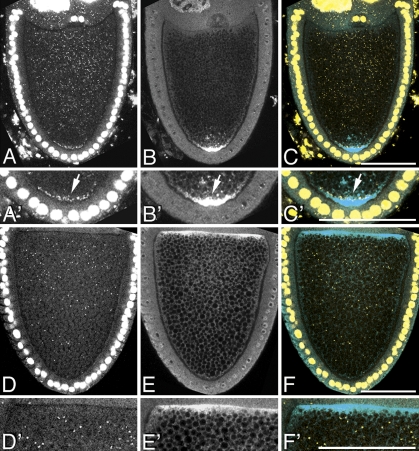
*wMel* in Oocytes That Exhibit Wild-Type and Ectopic Osk Localization (A–F) Full-size oocytes and (A'–F') corresponding expanded views of the posterior cortex are shown. Rows: (A–C, A'–C') *wMel* in a wild-type oocyte, (D–F, D'–F') *wMel* in an *osk^54^/oskDf^(3R)p-XT103^* oocyte carrying the *osk-bicoid 3'UTR* transgene. Columns: (A, A', D, D') propidium iodide stain, (B, B', E, E'), Osk antibody stain, (C, C', F, F') merged image showing propidium iodide (yellow) and Osk (cyan). Arrows indicate *wMel* and Osk co-localization. Scale bars = 25 μm.

**Figure 4:**
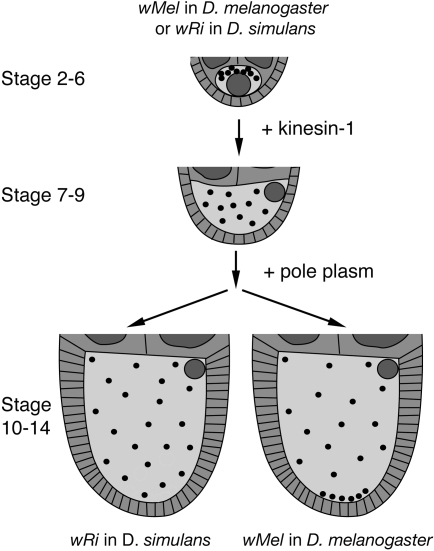
Model for Strain-Specific *Wolbachia* Localization Strategies *wMel* and *wRi Wolbachia* localize to the oocyte anterior from stages 3 to 6. Kinesin-1 transports *Wolbachia* away from the oocyte anterior during stages 7–9, carrying bacteria throughout the oocyte and toward the posterior pole. *wRi* remains evenly distributed into late oogenesis. In contrast, *wMel Wolbachia* near the posterior cortex interact with pole plasm to facilitate posterior *wMel* anchorage.

To test whether pole plasm is sufficient to drive *wMel* localization, we examined *wMel* in oocytes with anteriorly localized pole plasm. To this end, an *osk-bicoid 3'UTR* transgene was used to target *osk* mRNA to the oocyte anterior margin [38]. This ectopically localized *osk* is translated and assembles functional pole plasm at the antero-lateral cortex [38]. *wMel* co-localized with wild-type Osk protein at the oocyte posterior cortex (Figure 3A–3C, 3A'–3C'). However, *wMel* did not concentrate at the anterior margin with ectopically localized Osk in *osk-bicoid 3'UTR* oocytes (Figure 3D–3F, 3D'–3F'), suggesting that pole plasm alone is insufficient to recruit *wMel* from the cytoplasm. This result, taken together with those above, suggests that individual functions of kinesin-1 and pole plasm are both needed for robust posterior *wMel* localization in late stages 9 and 10A. This is consistent with a two-step mechanism for *wMel* localization: kinesin-1-mediated transport of *wMel* toward the oocyte posterior, followed by pole plasm-mediated anchorage of *wMel* to the posterior cortex (Figure 4).

### Factors Intrinsic to *Wolbachia* Are Needed for Posterior *Wolbachia* Localization

The extensive requirement of host components for posterior *wMel* concentration raises questions about whether *wMel* contributes to its localization. To investigate this, a trans-infection approach was employed using the host species, D. simulans, that normally carries the *wRi Wolbachia* strain [39]*.* In D. simulans oogenesis, *wRi* exhibited an anterior concentration during stages 3–6 and homogeneous distribution throughout the rest of oogenesis (Figure 1C, 1G, 1G', 1K, and 1K'; Table 1) [18]. Is this lack of posterior concentration due to differences between host oogenesis machinery or between the *wRi* and *wMel* strains? To address this, we examined D. simulans oocytes ectopically transformed with *wMel* [40]. *wMel*-infected D. simulans oocytes exhibited anterior *Wolbachia* concentration during early stages, homogeneous distribution in middle stages, and a striking posterior localization in late stages (Figure 1D, 1H, 1H' 1L, and 1L'; Table 1). This demonstrates that host components required for *Wolbachia* posterior localization are present in both D. melanogaster and D. simulans oocytes. Due to strain-specific differences, however, *wMel* engages those host components to enhance its posterior concentration in late oogenesis, whereas *wRi* does not.

Which oocyte components are engaged by *wMel* but not by *wRi*? Comparing *wMel* in *osk* mutant oocytes to *wRi* localization in D. simulans reveals a similar homogeneous distribution (Figures 1K and 2G). A speculative interpretation of this similarity is that *wMel* and *wRi* are similarly transported into the posterior half of the oocyte by kinesin-1. A further possibility is that *wRi* is unable to interact with host pole plasm, unlike *wMel*, which requires pole plasm for efficient posterior localization (Figure 2G and 2G'; Table 1). Perhaps unlike *wRi*, factors intrinsic to *wMel* drive interactions with posterior pole plasm that facilitate posterior *Wolbachia* anchorage (Figure 4).

## Discussion

### *Wolbachia* Localization Shares Some Common Features with Other Pathogens

The involvement of kinesin (this study) and dynein [18] in *Wolbachia* localization during oogenesis is reminiscent of microtubule-based transport employed by a number of human pathogens. Viruses such as herpes simplex virus type 1 rely on dynein and dynactin for their transport to a perinuclear position referred to as their “replication site” [41]. Kinesin transports the viruses back to the cell periphery, enabling their exit from the cell. Bacteria such as *Salmonella* are transported toward the host cell nucleus in a dynein/dynactin-dependent manner, which then facilitates bacterial replication [41]. *Salmonella* also actively recruits kinesin-1 to its surrounding membrane [42]. These observations suggest some parallels with *wMel*, which requires dynein and dynactin for anterior localization during early oogenesis [18] and kinesin-1 for posterior localization in late oogenesis. While the function of *Wolbachia* anterior localization is unclear, *Wolbachia* titer increases substantially at that location, suggesting that dynein-driven localization creates a replication site for *Wolbachia* within the oocyte [18]. Once replicated, kinesin-1-based transport enables *Wolbachia* to traverse the entire length of the growing oocyte, promoting *Wolbachia* incorporation into posterior pole cells. *Wolbachia* may therefore have sophisticated interactions with host motor proteins analogous to those used by other bacteria and viruses. The basis for a switch between dynein- and kinesin-1-dependent *Wolbachia* localization is currently unknown. In some systems the dynactin complex coordinates alternation of kinesin- and dynein-driven organelle motility [43]. Perhaps a regulatory agent like dynactin directs the changing *Wolbachia* localization pattern in oogenesis.

### Posterior *Wolbachia* Anchorage May Be a Cooperative Process

Upon reaching the posterior pole, *wMel* becomes anchored in a pole plasm–mediated manner. How might this occur? The simplest interpretation is that *wMel* associates directly with pole plasm components. However, a minority of *osk* null oocytes exhibited weak posterior *Wolbachia* localization (Table 1), although pole plasm is absent in this mutant background [38]. This suggests that other factors in addition to pole plasm assist posterior *Wolbachia* anchorage. Perhaps *wMel* has a dual affinity for pole plasm and an as-yet-unidentified posterior anchor. In such a case, the combined presence of those substrates may be important for robust *Wolbachia* anchorage to the posterior cortex. Alternatively, pole plasm may indirectly promote *Wolbachia* localization by stabilizing *Wolbachia* anchorage sites. A recent report indicated that Osk regulates actin polymerization at the oocyte posterior cortex [44]. It may be that *wMel* has a high affinity for unknown factors that associate with the posterior actin cortex, creating an indirect dependency of *wMel* upon posterior Osk.

One apparent conflict with these selective anchorage hypotheses is the finding that some colcemid- and colchicine-treated oocytes exhibit *Wolbachia* in association with the lateral cortex of the oocyte (Figure 2A). One interpretation of this result is that *Wolbachia* may have a general affinity for cortical actin independent of pole plasm. In such a scenario, one would predict that kinesin must normally drive *wMel* away from the lateral cortex and restrict it to the oocyte posterior where *wMel* is permitted to bind actin. This type of model has previously been proposed in the context of *osk* mRNA localization to the posterior pole [24,27]. If this prediction is accurate for *wMel* also, then oocytes lacking kinesin function should exhibit *wMel* localization to the antero-lateral cortex. However, *wMel* did not concentrate on the cortex of *Khc* null oocytes (Figure 2D). This suggests *wMel* does not have a general affinity for the actin cortex analagous to *osk* mRNA. An alternative interpretation of cortical *wMel* localization in colchicine- and colcemid-treated oocytes is that the drug treatments permitted microtubule remnants to remain along the cortex of some oocytes [21]. Those microtubule remnants could serve as a substrate for short-range *wMel* transport by kinesin-1, giving rise to a cortical *wMel* localization pattern. This possibility is consistent with the other findings of this study that favor kinesin-based *wMel* transport to the oocyte posterior, followed by selective *wMel* anchorage at the posterior pole.

### *Wolbachia* Localization Is Distinct from Other Factors in the Oocyte

The study presented here is one of the few to examine host–pathogen interactions in a developmental context. What emerges from this analysis is that the *Wolbachia* localization pattern is unique and does not follow specific morphogens or organelles during oogenesis. The *Wolbachia* localization pattern is distinct from mitochondria, which are concentrated on the posterior side of the oocyte nucleus during early stages, homogeneously distributed during mid-oogenesis, and posteriorly concentrated in stages 9 and 10 [45]. The anterior localization of *Wolbachia* precedes that of the determinant *bicoid* mRNA, which concentrates anteriorly from stages 6 to 14 of oogenesis [46]. *Wolbachia* posterior localization also appears later than *osk* mRNA, which concentrates posteriorly from stages 3 to 6, anteriorly in stage 8, and posteriorly again from stages 8 to 10 of oogenesis [47,48]. Furthermore, our study indicates that *Wolbachia* do not localize to the posterior cortex in association with *osk*/Stau mRNPs. Taken together, these observations suggest that the demands of replication and localization are unique to *Wolbachia* and may preclude these bacteria from hitchhiking on morphogens or organelles.

### Posterior Localization as an Adaptive Strategy for *Wolbachia*

The posterior localization strategy described in our report is exhibited by *Wolbachia* strains carried within multiple *Drosophila* and *Hymenopteran* species [1,9–11,14–17]. This recurrent localization pattern may reflect bacterial adaptations to the host environmental conditions. D. simulans allows *wRi* to persist at a high titer during embryogenesis, which is sufficient to promote *wRi* incorporation into posterior pole cells [10]. This environment may provide little incentive for *wRi* to evolve or retain a posterior localization strategy. The *wMel* strain, by contrast, is maintained at lower concentrations in D. melanogaster embryos [10]. This may pressure *wMel* to evolve and/or retain mechanisms that drive its posterior localization in oogenesis, thus enhancing its incorporation into embryonic pole cells. Taking advantage of kinesin-1 and pole plasm assembly at the oocyte posterior, as demonstrated by this study, provides an excellent means by which *Wolbachia* can accomplish this goal.

## Materials and Methods

### Fly strains.

*wMel Wolbachia* were crossed into wild-type D. melanogaster flies carrying the markers and balancers *w; Sp/Cyo, Sb/Tm6Hu*. This infected stock was used to cross *wMel* into all the D. melanogaster mutants used for this study, ensuring that all carried *wMel* strains of a comparable genetic background.

### Immunolabeling.

Ovaries were dissected and fixed using standard methods [23], then stained and imaged as previously [18]. Rabbit anti-Osk antibodies were used at 1:3000 [49]. Embryos were dechorionated with 50% bleach, fixed 20 min in a 1:1 mixture of 3.7% formaldehyde and heptane, and devitellinized by vigorous agitation in methanol. Embryos were stained with rabbit anti-Vasa at 1:2000 [50] and mouse anti-Hsp60 (Sigma) at 1:100 [18] in PBS/0.1% Triton, followed by 1:500 dilutions of Alexa-488- and Alexa-594-conjugated secondary antibodies (Molecular Probes).

### Microtubule inhibitor treatment.

Flies were starved 18 h, then fed 24–48 h with yeast paste containing 50 μM colcemid, 50 μM colchicine in DMSO, or comparable dilutions of DMSO alone. Mispositioning of the oocyte nucleus served as an internal control to verify that microtubule disruption had occurred [21,51].

### Microscopy and image analysis.

Images were acquired on a Leica DM IRB confocal microscope using a 63× oil objective and zoom factor of 1.5. Each oocyte was imaged as a z-series stack of 7–14 images spaced at 1.5-μm intervals. Optical sections deeper than 4.5 μm into the oocyte were examined for the presence of posterior *Wolbachia.* Oocytes were categorized in Table 1 as showing strong posterior localization if they exhibited striking *Wolbachia* staining, which consisted of either an intense linear array of *Wolbachia* puncta or a crescent-shaped area saturated with *Wolbachia* staining along the posterior cortex for four out of five consecutive z-sections. Oocytes were designated as showing weak posterior localization if they exhibited a.) at least one z-section with striking posterior localization, or b.) at least two z-sections with a higher *Wolbachia* density along the posterior cortex than in the cytoplasm of the cell. Oocytes were categorized as showing no posterior localization if they did not meet the above conditions. *Wolbachia* density was not analyzed in this study because oocytes carrying high bacterial loads exhibited saturation of *Wolbachia* labeling at the posterior pole that disrupted bacterial quantitation.

## Supporting Information

Figure S1 *wMel* and Pole Plasm Localization in Early Embryos Embryos are shown (A–C) prior to meiosis, (D–F) in cycle 11, (G–I) in cycle 14, and (J–L) during gastrulation. Posterior is facing down. Panel columns, left to right: (A, D, G, J) anti-Hsp60 staining indicating *wMel* [18,52], (B, E, H, K) anti-Vasa labeling pole plasm and pole cells [50], and (C, F, I, L) merged images showing anti-Hsp60 (yellow) and anti-Vasa (cyan). (A–C) At the beginning of embryogenesis, *wMel* is enriched at the posterior relative to the rest of the cortex (B, C). (D–F) *wMel* is incorporated into pole cells and (G–I) persists in pole cells as they multiply. (J–L) *wMel* is strongly concentrated in pole cells as they migrate into the embryo. Scale bar = 50 μm.
(6.3 MB TIF)

Figure S2 *Khc^27^* Oocyte Infected with *wMel*
Propidium iodide labeling of stage 5–6 oocytes shows anterior *wMel* localization. Scale bar = 25 μm.
(320 KB TIF)

### Accession Numbers

The NCBI Entrez (http://www.ncbi.nlm.nih.gov/sites/entrez?db=gene) accession numbers for the genes and gene products discussed in this paper are Hsp60 (P10809), Kinesin heavy chain (P17210), Oskar (P25158), Staufen (P25159), and Vasa (P09052).

## Abbreviations

*Khc*: *Kinesin heavy chain*
mRNP: messenger ribonucleoprotein particle
*osk*: *oskar*
*stau*: *staufen*
